# Characterization of conformational states of the homodimeric enzyme fluoroacetate dehalogenase by ^19^F–^13^C two-dimensional NMR†

**DOI:** 10.1039/d4cb00176a

**Published:** 2024-10-10

**Authors:** Motasem Suleiman, Geordon A. Frere, Ricarda Törner, Lauren Tabunar, Gaurav Vijay Bhole, Keith Taverner, Nobuyuki Tsuchimura, Dmitry Pichugin, Roman J. Lichtenecker, Oleksandr Vozny, Patrick Gunning, Haribabu Arthanari, Adnan Sljoka, Robert S. Prosser

**Affiliations:** a Department of Chemistry, University of Toronto UTM, 3359 Mississauga Rd Mississauga ON Canada L5L 1C6 scott.prosser@utoronto.ca; b Department of Biological Chemistry and Molecular Pharmacology, Harvard Medical School, Harvard University Boston USA; c Department of Cancer Biology, Dana-Farber Cancer Institute Boston USA; d Kwansei Gakuin University, Department of Informatics Nishinomiya 530-0012 Japan; e Institute of Organic Chemistry, University of Vienna Währingerstr 38 1090 Vienna Austria; f Department of Chemistry, University of Toronto, UTSC, EV 564 – Environmental Science & Chemistry 1065 Military Trail Scarborough ON Canada M1C 1A4; g RIKEN, Center for Advanced Intelligence Project 1-4-1 Nihombashi, Chuo-Ku Tokyo 103-0027 Japan adnan.sljoka@riken.jp; h Department of Biochemistry, University of Toronto, 1 King's College Circle, Medical Sciences Building Room 5207 Toronto ON Canada M5S 1A8

## Abstract

Tryptophan plays a critical role in proteins by contributing to stability, allostery, and catalysis. Using fluorine (^19^F) nuclear magnetic resonance (NMR), protein conformational dynamics and structure–activity relationships (SARs) can be studied *via* fluorotryptophan reporters. Tryptophan analogs such as 4-, 5-, 6-, or 7-fluorotryptophan can be routinely incorporated into proteins during heterologous expression by arresting endogenous tryptophan biosynthesis. Building upon the large ^19^F chemical shift dispersion associated with 5-fluorotryptophan, we introduce an approach to the incorporation of ^13^C-enriched 5-fluorotryptophan using a direct biosynthetic precursor, 5-fluoroanthranilic acid-(phenyl-^13^C_6_). The homodimeric enzyme fluoroacetate dehalogenase (FAcD), a thermophilic alpha/beta hydrolase responsible for the hydrolysis of a C–F bond in fluoroacetate, was expressed and biosynthetically labeled with (phenyl-^13^C_6_) 5-fluorotryptophan. The resulting two-dimensional ^19^F–^13^C (transverse relaxation optimized spectroscopy) TROSY heteronuclear correlation spectra provide complete resolution of all 9 tryptophan residues in the apo enzyme and FAcD saturated with the substrate analog bromoacetate. The (^19^F,^13^C) correlation spectra also reveal a multitude of minor resonances in the apo sample. The role of each tryptophan residue in allosteric communication was validated with computational rigidity transmission allostery analysis, which in this case explores the relative interprotomer communication between all possible tryptophan pairs.

## Abstract 2

5-Fluoroanthranilic acid-(phenyl-^13^C_6_) is derived from ^13^C-enriched 4-fluoronitrobenzene and converted to the tryptophan analogue, allowing (^19^F,^13^C) TROSY spectroscopy (created with BioRender.com).

## Graphical abstract

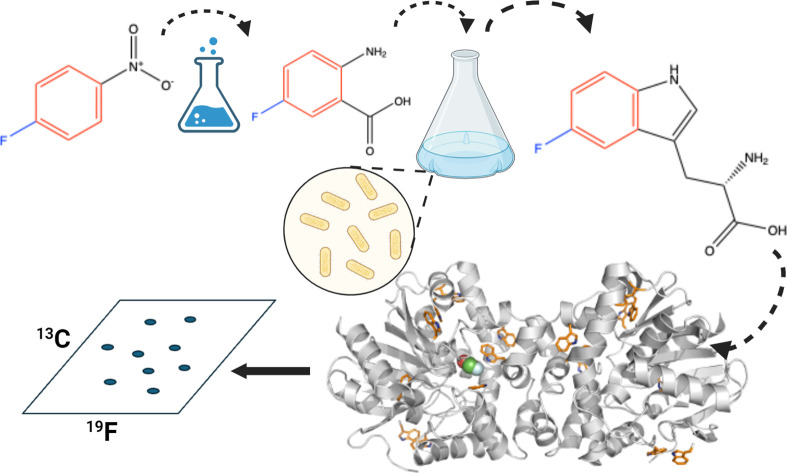

Owing to its exceptional sensitivity to electrostatic and van der Waals environments, fluorine (^19^F) NMR enjoys widespread usage in structure–activity relationship (SAR) studies aimed at understanding protein conformational dynamics and functional states.^1–9^ In cases where fluorinated amino acid analogs are used to characterize proteins, the vast majority are monofluorinated aromatic residues (tyrosine, phenylalanine, and tryptophan). Fluorinated tryptophan derivatives (particularly, 5-fluoro- and 7-fluorotryptophan) are nonperturbing and exhibit significant chemical shift dispersion.^10^ Until recently, however, much of ^19^F protein NMR has been confined to one-dimensional (1D) spectroscopy. By utilizing the 1-bond J_CF_ coupling of ∼250 Hz between aromatic ^19^F and ^13^C nuclei, heteronuclear ^19^F–^13^C two-dimensional (2D) NMR spectroscopy could improve overall resolution^11,12^ while allowing for more detailed dynamics studies that leverage single and double quantum coherences as well as ZZ-exchange experiments.^13^

Here, we present a method for the efficient biosynthetic incorporation of 5-fluoro-(phenyl-^13^C_6_)-l-tryptophan into any protein heterologously expressed in *E. coli*. In this scheme, 5-fluoro-(phenyl-^13^C_6_)-anthranilic acid is exogenously added to *E. coli* cell media during protein expression under induced tryptophan auxotrophy to yield ^13^C-enriched 5-fluoro-l-tryptophan. This opens the possibility of heteronuclear ^19^F–^13^C spectroscopy of tryptophan in proteins and may be expanded to other isotopically enriched tryptophan analogs derived from anthranilic acid.

While tryptophan abundance in proteins is 1.2% and 3.3% in soluble and membrane proteins, respectively, it plays a critical role in catalytic centers, stabilizing protein folds, and mediating allostery.^14^ The amphipathic indole side chain readily forms hydrogen bonds and engages in π-stacking with other aromatic residues (or oligonucleotides) or participates in highly stabilizing cation–π and anion–π interactions.^2,14^ In membrane proteins, the amphipathic nature of tryptophan is often utilized to anchor transmembrane domains to the membrane water interface of lipid bilayers.^15,16^

In *E. coli*, the incorporation of tryptophan analogs can be achieved by the direct addition of these analogs or their precursors as the corresponding indole or anthranilic acid derivatives.^17,18^ Recently, the synthesis of ^13^C-labeled 4-fluoro- and 7-fluoroindole was demonstrated, from which the tryptophan analog could be incorporated during expression in cell free media after supplying the media with ∼100 mg L^−1^ of the corresponding indole.^19^ Anthranilic acid analogs are a particularly attractive alternative since isotopically enriched variants can be readily synthesized from nitrobenzene and aniline starting compounds. Moreover, the biosynthetic pathway from anthranilate is directed exclusively to tryptophan in both *E. coli* and yeast, thus affording efficient biosynthetic labeling without dilution of the starting compound to off-pathway species *via* scrambling.^20^ Here, we use 5-fluoroanthranilic acid-(phenyl-^13^C_6_), a tryptophan precursor possessing a ^19^F–^13^C spin pair that can be biosynthetically incorporated directly into BL21 *E. coli* cell cultures at concentrations of ∼15 mg L^−1^ under induced tryptophan auxotrophy. Prior NMR studies using fluorinated tryptophan analogues have made use of either 4-, 5-, 6-, or 7-fluorotryptophan, supplied to the cell under glyphosate induced auxotrophy as either the amino acid or the indole precursor, using concentrations of 35–100 mg L^−1^.^10,17,19,21^ In our hands, we observe complete labeling, *via* fluoroanthranilate and induced auxotrophy at a fraction of these concentrations. We also observed that much higher concentrations of fluoroanthranilate actually compromised overall protein yield. To prepare the ^13^C-labeled tryptophan precursor, 4-fluoronitrobenzene-^13^C_6_ was hydrogenated to afford the corresponding aniline hydrochloride which was subsequently converted into 5-fluoroisatin-^13^C_6_*via* the Sandmeyer procedure (Scheme 1). Oxidative decarbonylation of this material produced the target molecule 5-fluoroanthranilic acid-(phenyl-^13^C_6_). In addition to its high solubility, anthranilic acid derivatives have the added advantage that these precursors yield the corresponding tryptophan analogs in both *E. coli* and yeast expression systems. There may also be a wide range of anthranilate analogs of potential value in studying enzymology, metabolic flux profiling, or as therapeutics. Interestingly, 4-fluoroanthranilate has been shown to impair growth in *M. tuberculosis*, suggesting a motivation for exploring additional anthranilic acid derivatives as potential antibiotics.^22^

**Scheme 1:**
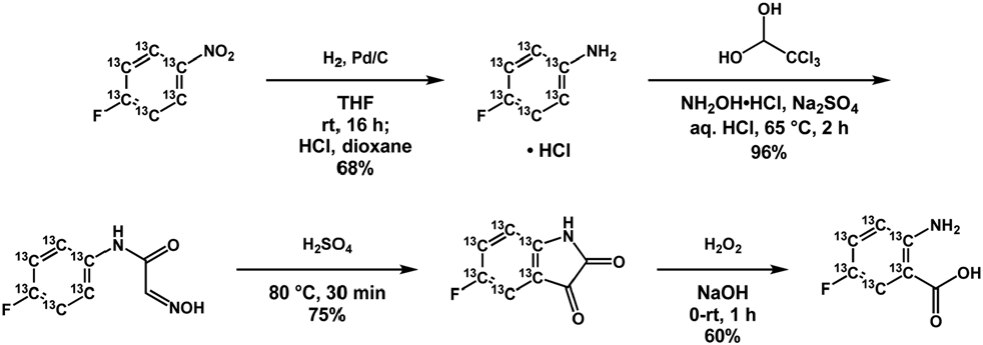
Synthesis of 5-fluoroanthranilic acid-(phenyl-^13^C_6_) from 4-fluoronitrobenzene-^13^C_6_.

To demonstrate the application of 5-fluoro-(phenyl-^13^C_6_)-l-tryptophan as an NMR reporter, we expressed the 300-residue homodimeric enzyme fluoroacetate dehalogenase (FAcD) using 5-fluoroanthranilic acid-(phenyl-^13^C_6_) as a metabolic precursor. Prior to induction, cells were treated with glyphosate to halt aromatic synthesis, while the cell culture was simultaneously supplied with tyrosine, phenylalanine, and the ^13^C-enriched fluoroanthranilate. Fluoroanthranilate then rescues tryptophan synthesis and hence biosynthetic enrichment of the target protein with 5-fluoro-(phenyl-^13^C_6_)-l-tryptophan.

FAcD is an alpha/beta hydrolase responsible for the hydrolysis of the C–F bond in its substrate fluoroacetate.^21,23^ Each protomer contains 9 tryptophan residues, two of which (Trp156 and Trp185) are situated in the active site pocket (Fig. 1). FAcD undergoes strict half-of-sites reactivity, suggesting that the NMR signatures of Trp156 and Trp185 should be differentiated in each protomer, upon binding substrate.^23^ However, binding of substrate initiates a pronounced allosteric response, resulting in local disorder in the empty protomer and rapid interprotomer dynamics such that there is generally no protomer-distinct signal.^21,23^ Conveniently, the substrate analog bromoacetate (BrAc) is not reacted and can therefore be used to study the Michaelis–Menten intermediate and associated dynamics.^23^

**Fig. 1:**
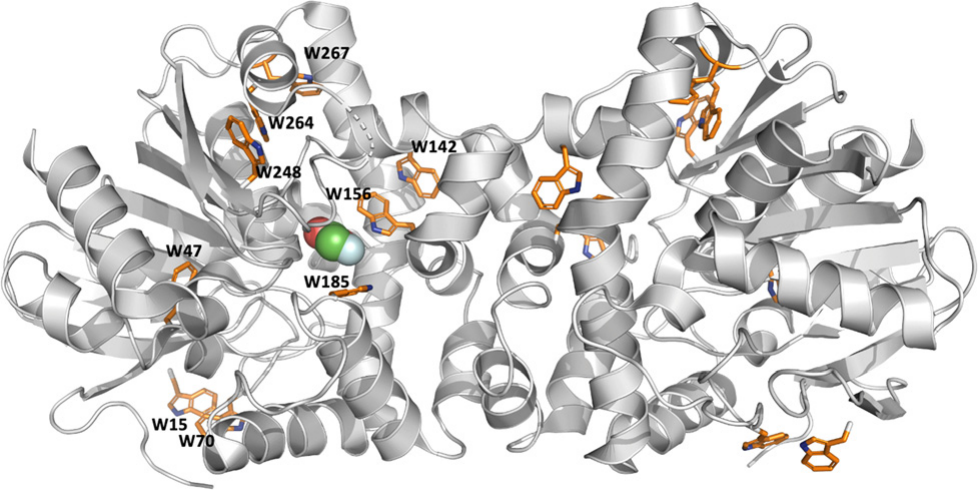
Structure of the FAc-bound FAcD dimer (5SWN) illustrating the Trp side chains. Of note, W156, W185, and W142 lie in or around the active site pocket.

Two-dimensional (2D) ^19^F–^13^C transverse relaxation optimized spectroscopy (TROSY) heteronuclear correlation spectra of apo FAcD and BrAc-bound FAcD, were acquired as previously described.^12^ Overlays of the 2D contours are shown in Fig. 2. Assignments were obtained through mutagenesis and 1D ^19^F NMR. Note that the additional ^13^C dimension in the 2D spectrum completely resolves the central cluster of ^19^F-resonances arising from W264 and W267 at −123.75 ppm (^19^F) in addition to three otherwise severely overlapped resonances (W15, W70, and W142) around −124.75 ppm in the apo protein at 60 °C (Fig. 2A). The addition of BrAc (blue contours, Fig. 2B) results in significant exchange broadening of W156 in the active site pocket^21,23^ such that the resulting resonance (expected between −119 and −118 ppm in the ^19^F dimension) is not observed in the 2D NMR spectrum. The corresponding 1D ^19^F NMR spectrum, obtained from a separate equivalent sample, is overlayed in Fig. 2B, revealing the exchange-broadened ^19^F NMR resonance of W156. The addition of BrAc is also observed to result in a pronounced shift for W185, situated in the substrate pocket, as shown in Fig. 1. Clear shift perturbations are also observed for the peaks associated with W264 and W267 after addition of BrAc. The current ^19^F–^13^C TROSY HSQC spectra were acquired with the use of band-selective homonuclear ^13^C-refocusing pulse as discussed in the Methods section, effectively removing the effects of the ^13^C–^13^C one-bond coupling. The ^13^C–^19^F 1-bond scalar coupling is ∼250 Hz, allowing for very short INEPT transfer periods (∼1.7 ms) after balancing losses due to T_2_-relaxation with optimal INEPT transfer.

**Fig. 2:**
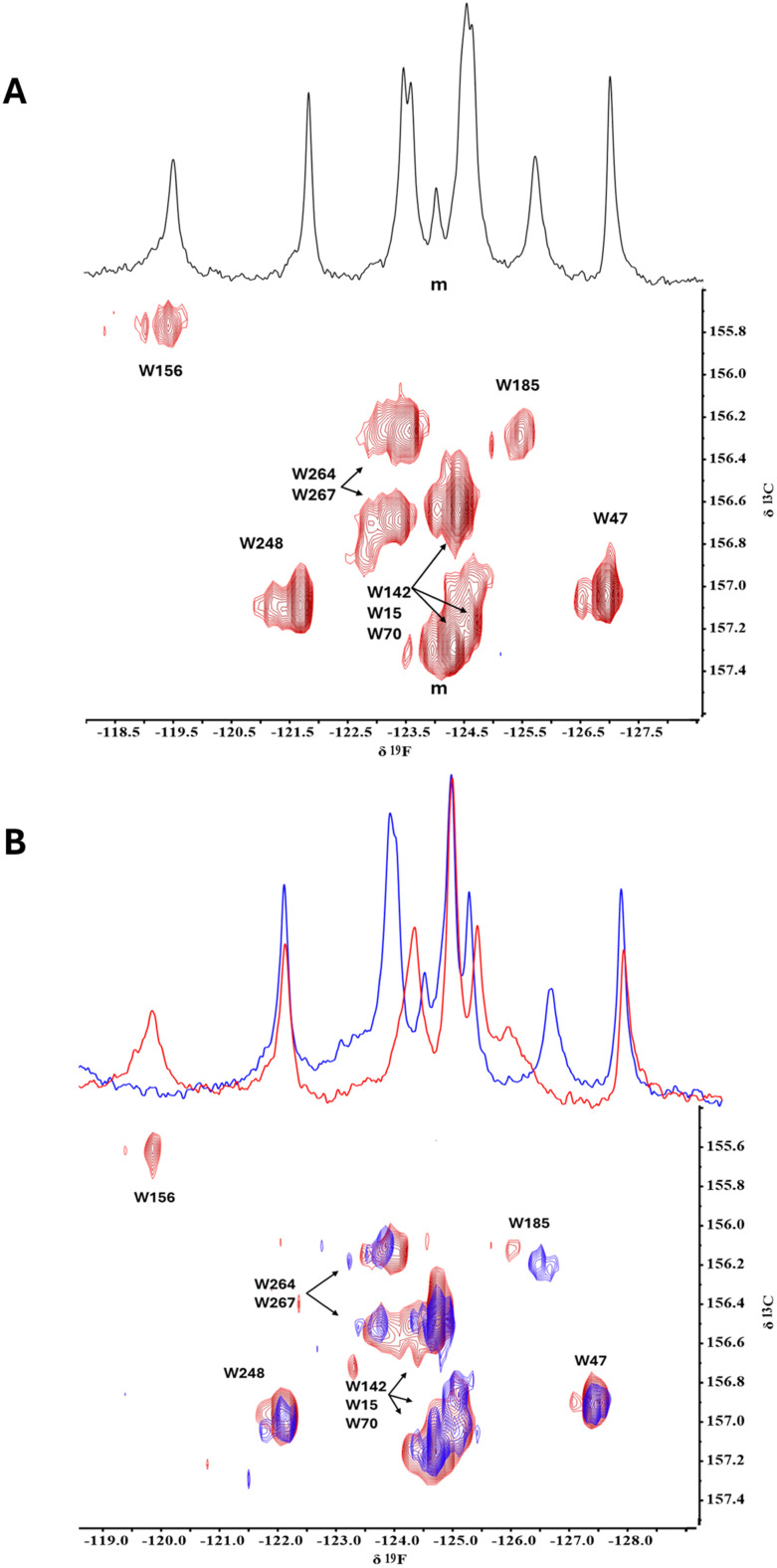
(^19^F,^13^C) TROSY HSQC spectra of apo FAcD (red) and BrAc-bound FAcD (blue). (A) TROSY spectrum of apo FAcD at 60 °C. Partial assignments were obtained by mutagenesis. A prominent minor peak, designated as ‘m’, is also shown in the 1D and 2D spectra. (B) TROSY spectra of apo FAcD (red) and BrAc-bound FAcD (blue) at 50 °C. The ^19^F 1D NMR spectra at the top were acquired separately and are added as a visual comparison.

FAcD is found in a thermophilic organism and under the buffer and pH conditions used in these experiments, can be reconstituted at 200 μM concentrations and 60 °C for 24–48 hours before the onset of gradual precipitation. In the ^19^F–^13^C 2D TROSY spectrum of apo FacD at 60 °C, shown in Fig. 2A, a dramatic improvement is observed in resolution, where an additional subset of minor peaks is observed for the majority of resonances.^21^ Interestingly, the minor peak associated with Trp47 and the single sharp resonance, designated as ‘*m*’ in Fig. 2A, are consistent with shift perturbations previously seen with a K152I mutant, used to explore a second substrate binding site.^23^ In this earlier work, the authors proposed that the substrate first binds to an outer pocket near K152, where it is desolvated prior to diffusing into the active site pocket. It is possible that these minor peaks are therefore associated with a substrate bound like state, suggested earlier based on a limited CEST study on Trp156 by 1D ^19^F NMR.^21^ The minor peak designated as “*m*” in the 1D ^19^F NMR spectrum of apo FAcD at 60 °C, integrates to half that of the other single resonances, implying that the protomer-specific chemical shift signatures are nondegenerate. This peak also overlaps with the (W15, W70, W142) cluster and most likely originates from W142, which is thought to be situated along a pronounced allosteric pathway in the Michaelis–Menten intermediate.^21,24^ This same peak can also be observed at 50 °C upon addition of BrAc (Fig. 2B), where protomer asymmetry is expected to be greatest. In the 2D NMR, many minor state resonances now become well resolved. Such slow exchange dynamics could in future be studied using a ^19^F–^13^C TROSY selected ZZ-exchange experiment.^25^ However, for the moment, these longer experiments are limited by lifetime of the sample.

Using rigidity transmission allostery (RTA), it is possible to examine the role of the tryptophan sidechains throughout the dimer in facilitating cooperative dynamics upon substrate binding.^21,23,26–30^ In many cases, residue specific-chemical shift changes arising from addition of a substrate analog, often correlate with allosteric propensity determined *via* RTA.^21^ In our case, any tryptophan residues that belong to an allosteric activation network should exhibit greater ^19^F chemical shift perturbations with titration of BrAc. Substrate binding engages the W156 sidechain *via* a hydrogen bond between the halogen and the indole nitrogen, while additionally restricting W185 in the bound protomer (Fig. 1). However, this initiates a prominent allosteric response in the empty protomer and the onset of significant interprotomer dynamics.^21,23^ RTA methods furnish a mechanistic model of allosteric communication by analyzing how local perturbations of rigidity and conformational degrees of freedom propagate from one site to modify rigidity at distant sites across the complex. Building on previous work, here we extend RTA analysis and examine all possible interprotomer pairwise tryptophan allosteric interactions (W_*i*_, W_*j*_).

In the current study, each tryptophan residue and all contacts within a 3 Å radius were separately rigidified in the empty protomer and the resulting change in conformational degrees of freedom in the bound (second) protomer were then assessed. This prediction allows us to assess the magnitude of interprotomer allosteric response between tryptophan residues. Note that in this computational approach the results are symmetric, meaning that we could in principle begin with either the bound or free protomer in investigating the magnitude of interprotomer response. This response is shown in Fig. 3 as a heat map. W156, W185, and W142 all show a pronounced interprotomer allosteric connectivity. Note that W264 and W267 are also somewhat more weakly connected to this core of allosterically coupled residues. In the absence of substrate, the allosteric connectivities are predicted to be considerably weaker, as shown in Fig. 3.^8,14^ The slight asymmetry in the heat map is due to subtle asymmetry in structure of the two protomers.

**Fig. 3:**
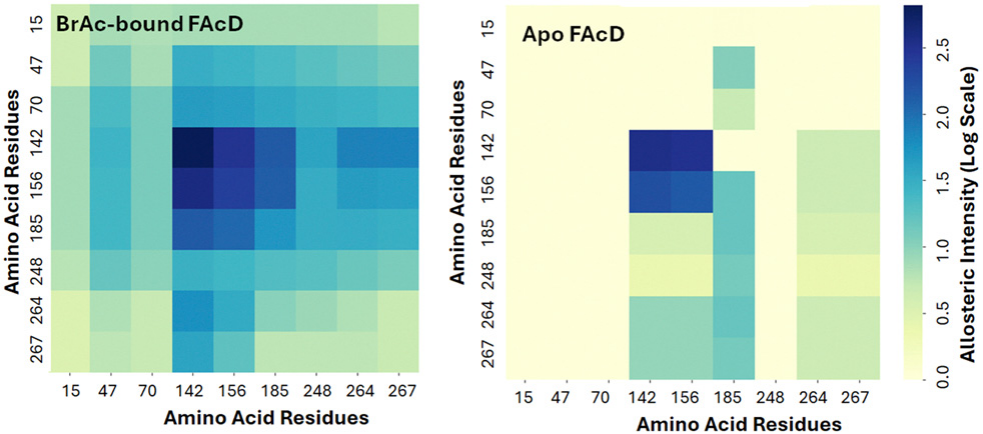
RTA interprotomer heat map showing allosteric coupling between each Trp residue in the empty protomer to the Trp residues in the BrAc-bound protomer. Interprotomer allostery is considerably weaker in the apo structure.

Lastly, we can explore the extent to which each tryptophan residue contributes to overall interprotomer allostery based on RTA and then map this response on the protein surface, as shown in Fig. 4. Note that the interprotomer allosteric pathways arising from perturbation of either W142, W156, or W185, are all intense and reveal a similar network. In all cases, the residues associated with interprotomer allosteric response overlap and are largely common to the allosteric network resulting from perturbation of the substrate pocket.^21,23^ In contrast, perturbation of W47 reveals a relatively weak interprotomer response.

**Fig. 4:**
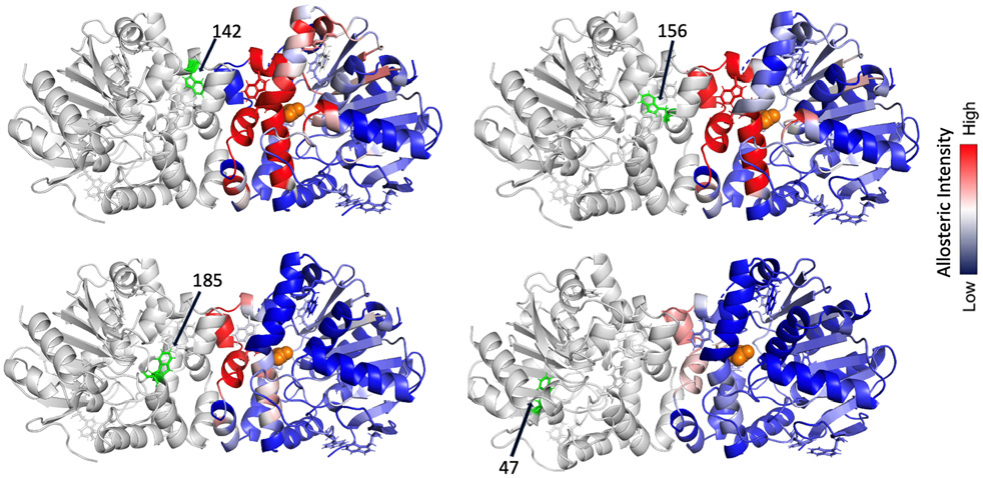
RTA maps predicting the interprotomer allosteric network resulting from perturbing each Trp residue in the opposite protomer, using the BrAc-bound FAcD crystal structure. Perturbation of W142, W156, and W185 results in similar and intense responses in allosteric transmission in large regions of the opposite protomer, while residues that are not a part of the allosteric network (*e.g.*, W47) show a very weak overall response.

To explore the utility of deuteration in fluoro-tryptophan analogs, we also synthesized 5-fluoroanthranilic acid-d_3_ from fluorobenzene-d_5_ using a protocol analogous to that shown in Scheme 1 with the addition of an initial nitration step. In this case, we anticipated improvements in ^19^F NMR linewidths through removal of intramolecular ^1^H–^19^F dipolar relaxation. However, in the case of FAcD even at temperatures exceeding 50 °C where some ^19^F linewidths reached 60–75 Hz, deuteration of fluoroindole side chains imparted no benefit in ^19^F linewidths (data not shown). However, deuteration would be expected to improve transverse relaxation, particularly in the ^13^C evolution period in a (^19^F,^13^C) TROSY. Recently, Toscano *et al.* published an organic scheme for the synthesis of ^13^C/^19^F/^2^H phenylalanine analogs.^31^ Excitingly, their scheme describes an affordable 4-step synthetic route to 4-fluoro, 4-^13^C, 3,5-deutero aniline, which is a critical precursor in Scheme 1, described above. In this case, the full potential of the ^19^F,^13^C TROSY could be exploited, without the inconvenience of ^13^C–^13^C-decoupling and 2-bond ^13^C–^1^H couplings, which compromised the current TROSY spectra.

In this work, we have demonstrated the straightforward synthesis of ^13^C-enriched fluorinated anthranilic acid analogs, which give rise to corresponding tryptophan analogs upon their introduction in the *E. coli* expression system, without scrambling. The 2D ^19^F,^13^C TROSY HSQC spectra proved adequate for resolution of all 9 tryptophan residues, revealing an interprotomer allosteric network employing multiple tryptophan residues. At higher temperatures, a possible substrate-bound like state is observed in slow exchange with the ground state of the apo protein. Using a different labeling scheme to generate 7-^13^C,7-^19^F-indole, preliminary experiments by Maleckis *et al.* suggest TROSY improvements in ^13^C transverse relaxation could be on the order of a factor of three in a ^19^F,^13^C TROSY experiment,^19^ while the recent work by Toscano offer the means to isolate the ^19^F,^13^C pair in 5-fluoro-tryptophan from homonuclear ^13^C–^13^C couplings and two bond ^13^C–^1^H couplings and relaxation effects.

## Author contributions

The manuscript was written through contributions of all authors. All authors have given approval to the final version of the manuscript.

## Conflicts of interest

There are no conflicts to declare.

## Supplementary Material

CB-005-D4CB00176A-s001

## Data Availability

The data supporting this article have been included as part of the ESI.† Original raw NMR data (1D and 2D NMR) are available to readers by contacting the corresponding authors. All processing software is described in the ESI.†
