# Supplementary material for: Characterization of conformational states of the homodimeric enzyme fluoroacetate dehalogenase by 19F–13C two-dimensional NMR
Source: RSC Chem Biol. 2024 Oct 10;5(12):1214–8. doi: 10.1039/d4cb00176a (PMC11465415; doi:10.1039/d4cb00176a)
Supplement: CB-005-D4CB00176A-s001 [file CB-005-D4CB00176A-s001.pdf]

Supplementary Information (SI) for RSC Chemical Biology.
This journal is © The Royal Society of Chemistry 2024

Supplementary Information

Characterization of Conformational States of the Homodimeric Enzyme Fluoroacetate Dehalogenase by ^{19}F-^{13}C Two-Dimensional NMR

Motasem Suleiman[1‡], Geordon A. Frere[1 ‡], Ricarda Törner [2,3], Gaurav Vijay Bhole[2,3], Keith Taverner[1], Nobuyuki Tsuchimura[4], Lauren Tabunar[1], Dmitry Pichugin[1], Roman J. Lichtenecker[5], Oleksandr Vozny[6], Patrick Gunning[1], Haribabu Arthanari[2,3], Adnan Sljoka[7*], and Robert S. Prosser[1,8*]

[1]Department of Chemistry, University of Toronto, UTM, 3359 Mississauga Rd, Mississauga, ON, Canada L5L 1C6. [2]Department of Biological Chemistry and Molecular Pharmacology, Harvard Medical School, Harvard University, Boston, USA. [3] Department of Cancer Biology, Dana-Farber Cancer Institute, Boston, USA. [4]Kwansei Gakuin University, Department of Informatics, Nishinomiya, 530-0012, Japan.[5]Institute of Organic Chemistry, University of Vienna, Währingerstr 38, 1090 Vienna, Austria. [6]Department of Chemistry, University of Toronto, UTSC, EV 564 - Environmental Science & Chemistry, 1065 Military Trail, Scarborough, ON, Canada M1C 1A4. [7]RIKEN, Center for Advanced Intelligence Project, 1-4-1 Nihombashi, Chuo-Ku, Tokyo 103-0027, Japan.[8]Department of Biochemistry, University of Toronto, 1 King's College Circle, Medical Sciences Building, Room 5207, Toronto, ON, Canada M5S 1A8.

[‡] These authors contributed equally to this work

[*] Corresponding authors

scott.prosser@utoronto.ca

adnan.sljoka@riken.jp

mailing address: Chemical and Physical Sciences, UTM

3359 Mississauga Rd,

Mississauga, ON

L5L 1C6

Canada

Section S1. General information

All solvents and chemicals were used as purchased without further purification. Reactions were carried out in oven-dried glassware and monitored by thin-layer chromatography (TLC) using Merck silica gel 60F254 on aluminum sheets (visualized by 254/365 nm UV light and/or staining with ninhydrin). Column chromatography was carried out using Biotage Isolera One and Isolera

Prime purification systems, with industry-standard Sfar Duo 10, 25, 50, and 100 g cartridges loaded with 40–60 μm silica gel from VWR International (average pore size, 60 Å) eluting at 12–40 mL/min and detecting compounds by UV measurements at 254 and 298 nm. Mass spectrometry (MS) was carried out using a Waters LC–MS in ESI mode fitted with a Micromass ZQ MS and an Alliance 2690 LC. Analysis was performed on a Waters Xterra C18 3.0 mm × 150 mm column containing 3.5 μm beads by direct injection into the mass spectrometer.

Expression, and Purification of FAcD

FAcD from *Rhodopseudomonas palustris* was expressed in *Escherichia coli* BL21 (DE3) Star Cells and purified as reported elsewhere with some modifications[1,2]. For the preparation of NMR samples, a single colony was inoculated in 100 mL LB starter culture at 37 ºC for approximately 10 hours. The cells were pelleted at 6000 g for 10 minutes and resuspended in 100 mL of M9 media. The cells were left to grow overnight and were transferred to 900 mL M9 media in a 1-liter flask. The cells were grown to OD_{600} of 0.6 whereupon 1 g of glyphosate, 0.075 g of tyrosine, 0.075 g of phenylalanine, and 0.005 g of 5-fluoroanthranilic acid-(*phenyl*-$^{13}C_6$) were added. Glyphosate halts the synthesis of aromatic amino acids and allows the cells to convert 5-fluoroanthranilic acid-(*phenyl*-$^{13}C_6$) to 5-fluorotryptophan-(*phenyl*-$^{13}C_6$) before induction. Once OD_{600} reached 0.8-1.0, cells were induced with 2 mM isopropyl *β*-D-1-thiogalactopyranoside (IPTG) for expression for 4 hours. *E. coli* cells were pelleted and were resuspended using a lysis buffer composed of 50 mM HEPES, pH 7.5, 500 mM NaCl, 5% glycerol, and 5 mM imidazole. The cells were lysed via an LM-20 microfluidizer followed by ultracentrifugation to obtain the cell-free lysate. The lysate was then loaded onto a Ni-NTA His-binding resin, which was washed with lysis buffer with increasing the concentration of imidazole to 20 mM. The same buffer was used for elution at 20 mM imidazole. The eluent was placed in a 3.5 kDa MW cut-off dialysis bag containing 0.6 mg/mL TEV protease. The cleavage of the His_6-tag was performed in 50 mM Tris-HCl and 1 mM DTT at room temperature for 4 hours. The protein was further purified by size exclusion chromatography (HiLoad 16/600 Superdex 200 prep grade size exclusion column) that was equilibrated with 50 mM Tris-H_2SO_4 at pH 8.5. FAcD was stored in the same buffer at room temperature or 4 C prior to the NMR experiment.

^{19}F 1D and ^{19}F,^{13}C 2D NMR

^{19}F 1-dimensional (1D) NMR spectra were obtained at 50 °C and 60 °C using a 600 MHz Varian Inova spectrometer equipped with a^{19}F-tunable HCN probe. ^{19}F NMR spectra were recorded with FAcD enriched in 5-fluorotryptophan alone. 1D ^{19}F NMR spectra were processed using exponential apodization (lb=25 Hz), and backwards linear prediction by 5 points using the Toeplitz method. Using otherwise identically prepared samples, ^{13}C-decoupled ^{19}F NMR 2D NMR spectra were recorded with FAcD enriched in 5-fluorotryptophan-(*phenyl*-$^{13}C_6$), using a Bruker Avance III HD console and a QCI-F cryoprobe and z-shielded gradients. The NMR titration sample was prepared by adding a stock solution of BrAc (in 1:30 molar ratio) to the 300 μL of 5-fluoro-tryptophan-enriched FAcD sample at a concentration of 265 μM. All spectra were collected with ^{19}F detection centered at -123 ppm (SW:40 ppm) and ^{13}C-detection centered at 157 ppm. The number of scans was increased at high concentrations of titrant to observe exchange broadened peaks. We quantified the protein concentration on a NanoDrop instrument by measuring the absorbance at 280 nm (A_{280}), assuming an extinction coefficient of 65890 M^{-1} cm^{-1}. Additionally, ^{19}F NMR spectra of FAcD were then recorded in the presence of a known concentration of a trifluoroacetic acid (TFA) standard. After measuring the area under the fluorotryptophan peaks relative to that of the standard, we established that the protein was uniformly labeled at a level close to 90%.

We recoded the ^{19}F-^{13}C correlated 2D experiments through a traditional 2D-FC-HSQC out-and-back spectrum with TROSY selection, modified from the standard Bruker pulse sequence "hsqcph".[3,4] To refocus the homonuclear ^{13}C-^{13}C coupling we employed a selective homonuclear carbon-refocusing pulse generated by optimal control theory (OCT) and detailed description of the pulse design is given below. This homonuclear decoupling pulse selectively inverts (unitary rotor) carbon resonances in the range of 100 to 135 ppm while preserving the desired resonances from 155 ppm to 165 ppm, i.e. the magnetization in the desired bandwidth returns to the same position as it was before the start of the pulse. This homonuclear decoupling pulse does not require a Bloch Siegert Shift compensation. The TROSY experiments were collected using an out-and-back (starting with ^{19}F magnetization and detecting ^{19}F) strategy. The INEPT transfer times were set based on a ^{19}F-^{13}C coupling of 240 Hz. 128 complex points were collected in the indirect dimension with a recycling delay of 1 sec. Data were processed using Mnova software, that

included truncation of the original data to 300 points (50°C data) or 350 points (60°C data) prior to zero filling and exponential apodization (lb=10 Hz), while the indirect dimension was processed using a 90 degree shifted sine-squared apodization filter and zero filling.

Design of the optimal control ^{13}C-^{13}C homonuclear decoupling pulse: We designed the refocusing pulse using a modified version of a popular optimal control algorithm, GRAPE (Gradient Ascent Pulse Engineering) as described in (Seedless: On-the-fly pulse calculation for NMR experiments, Charles Buchanan et al., bioRxiv 2024.01.31.578133). We use a typical 'piecewise' constant ansatz for the control variables (amplitude and phase in our case) with a total of 300 segments of 2 μs each. Further, the amplitude across all segments was held constant at 16.67 kHz (equivalent to a 90-degree hard pulse of duration 15 μs), while allowing only for phase modulation. About 30 uncoupled spins were uniformly sampled from each band and the combined cost function for optimization was given by their ensemble averaged fidelity. The real part of the Hilbert-Schmidt inner product of the obtained unitary evolution and the target unitary operator was chosen to be the fidelity function. The target operator for band A (100-135 ppm) was a unitary 180-degree rotation about the x-axis while the target for band B (155-165 ppm) was the 2x2 identity matrix. A Broyden–Fletcher–Goldfarb–Shanno (BFGS) optimization algorithm was employed through the inbuilt MATLAB routine fminunc. Since the closed system dynamics of a single isolated spin can be evaluated analytically, we use exact closed form expressions for the piecewise unitary propagators as well as their gradients, which greatly improves the computational speed as well as the convergence of BFGS. The behavior of the pulse in the desired encoding bandwidth and the bandwidth to be decoupled is shown in Supplementary Figure S1.

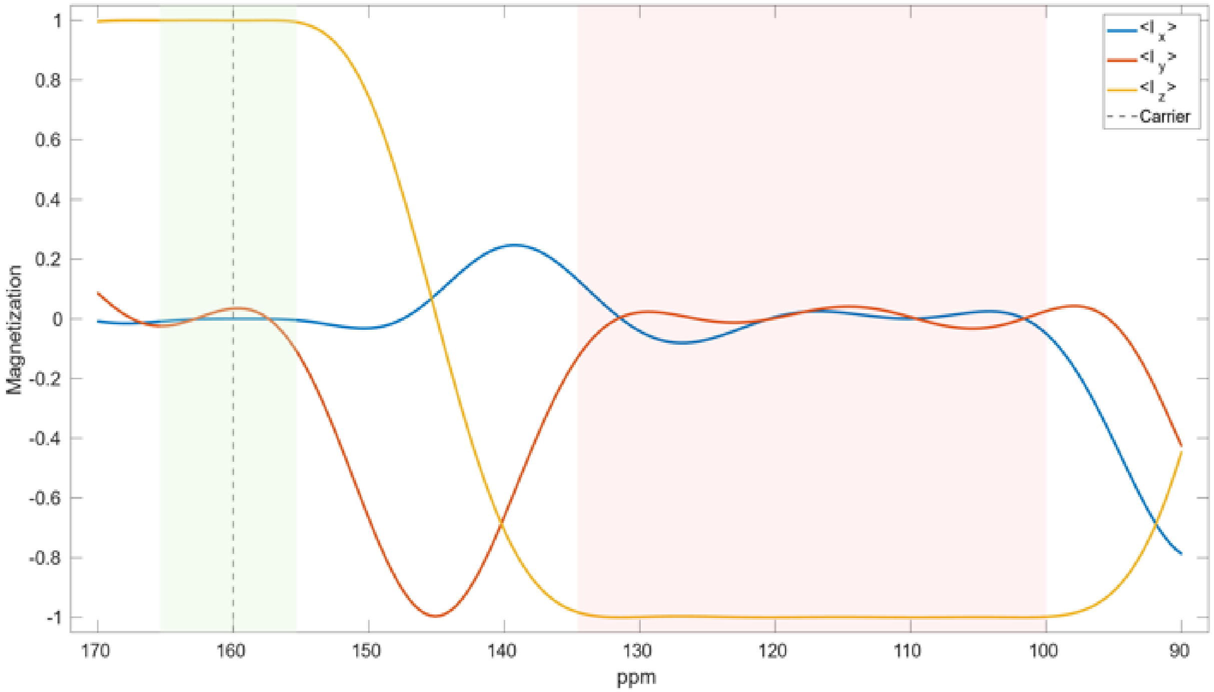

Figure S1 Simulated magnetization plots for the designed optimal control pulse. The starting magnetization of all spins is taken to be in the state I_z. The pulse is designed to implement a unitary 180-degree rotation about the x-axis in the 100-135 ppm band (transparent red block) while implementing an identity operation between 155 and 165 ppm (transparent green). The blue, orange and yellow curves are for the Uhlmann-Jozsa fidelity between the evolved density matrix starting from state I_z and final states I_x, I_y and I_z respectively.

Computational procedures

Rigidity Transmission Allostery (RTA).

The allosteric networks in apo and BrAc-bound FAcD crystal structures were analyzed using Rigidity Transmission Allostery (RTA) theory and algorithms.[5–9] RTA - a computational method based on mathematical rigidity theory[10,11] – seeks to predict and quantify if perturbations in conformational rigidity and flexibility (i.e., degrees of freedom) at one region (site) on the protein structure can propagate and affect degrees of freedom in other regions of protein structure. For both apo and substrate bound FAcD dimer structures, we quantified the extent of allosteric crosstalk between the two monomers originating at every tryptophan in an empty monomer. We used the method FIRST[12] to construct a constrained network (graph) for both apo and substrate bound structures. In this graph, vertices are atoms and edges model covalent and non-covalent

interactions. We independently rigidified each tryptophan residue (plus 3 Å radius sphere around its atoms) in empty monomer and quantified the resulting change in degrees of freedom (i.e. allosteric intensity) for each residue in the second monomer. This was repeated for different hydrogen bond energy cutoffs by iteratively removing weak hydrogen bonds one by one from the structure. To visualize the allosteric influence of one tryptophan residue on all other residues in the empty monomer, residues were color-coded on the 3D structure based on the extent of allosteric transmission. Residues exhibiting high allosteric intensity transmission delineate the allosteric pathways. To indicate the allosteric influence of each tryptophan on other tryptophan residues in the second monomer, a heatmap was used to indicate their pairwise allosteric communication strength. A log scale of allosteric strength was used in a heatmap of tryptophan-tryptophan allosteric coupling.

Section S2. Synthetic procedures

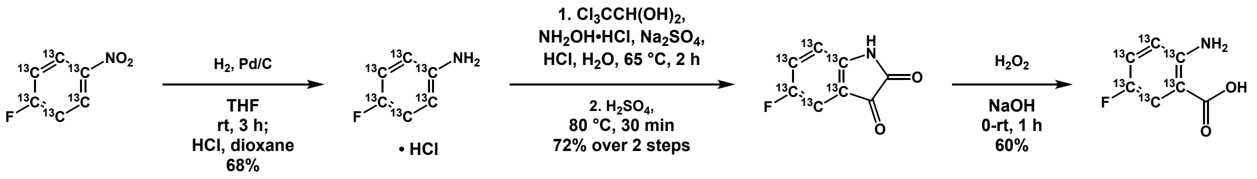

Scheme S1. Synthetic scheme for 5-fluoroanthranilic acid-(*phenyl*-$^{13}C_6$).

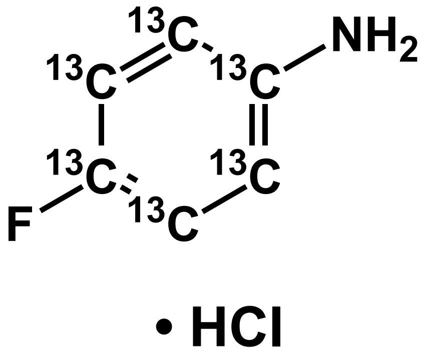

4-Fluoroaniline-$^{13}C_6$ hydrochloride

10% palladium on carbon (362 mg, 0.34 mmol, 10 mol%) was added to an oven-dried, nitrogen-purged round-bottom flask containing a magnetic stir bar. The palladium was wetted with minimal THF, and the flask was purged three times with nitrogen. A solution of 4-fluoronitrobenzene-$^{13}C_6$ (500 mg, 3.4 mmol, 1.0 equiv.) in THF (17 mL, 5 mL/mmol) was added carefully with stirring. Hydrogen was bubbled through the stirring reaction mixture for 16 hours. Upon complete consumption of starting material by TLC, the reaction was filtered through a celite plug and washed three times with THF. Hydrogen chloride (4 M in 1,4-dioxane; 0.85 mL, 1.0 equiv.) was added dropwise to the filtrate and fine, off-white crystals precipitated from the solution. The mixture was diluted with anhydrous diethyl ether and the precipitate was collected by vacuum filtration to afford the product as a colorless, crystalline solid (372 mg, 2.42 mmol, 68% yield). ^{1}H NMR (400 MHz, D_2O) δ 7.62 – 7.06 (m, 1H), 7.46 – 6.85 (m, 1H). ^{13}C NMR (101 MHz, D_2O) δ 162.29 (dtd, J = 246.0, 71.8, 10.2 Hz), 127.69 – 123.50 (m), 118.62 – 115.63 (m). ^{19}F NMR (376 MHz, D_2O) δ -112.61 – -113.70 (m). MS (ESI+): 117.96.

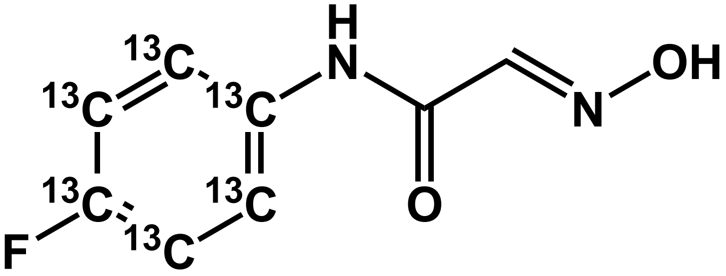

N-(4-Fluorophenyl)-2-isonitrosoacetanilide-(*phenyl*-$^{13}C_6$)

To a three-necked flask equipped with a thermometer, chloral hydrate (425 mg, 2.57 mmol, 1.1 equiv.) was dissolved in water (11.7 mL, 5 mL/mmol) and the solution was stirred until chloral hydrate dissolved completely. Anhydrous sodium sulfate (6.08 g, 42.8 mmol, 18.3 equiv.) was added slowly into the mixture with vigorous stirring, followed by hydroxylamine hydrochloride (488 mg, 7.02 mmol, 3.0 equiv.). 4-Fluoroaniline-$^{13}C_6$ hydrochloride (359 mg, 2.34 mmol, 1.0 equiv.) and concentrated hydrochloric acid (0.103 mL, 0.044 mL/mmol) were sequentially added. This solution was added to the first solution in one portion, resulting in a milky white solution, and the reaction mixture was heated to 65 °C and stirred for 2 h until TLC revealed complete consumption of starting material. The resulting precipitate was collected by filtration and washed with water. An off-white, crystalline powder was obtained after drying overnight in a vacuum desiccator over phosphorus pentoxide and was used directly in the next step without further purification (420 mg, 2.23 mmol, 96% yield). ^{1}H NMR (400 MHz, CD_3OD) δ 7.95 – 7.37 (m, 1H), 7.60 (s, 0H), 7.36 – 6.81 (m, 1H). ^{13}C NMR (101 MHz, CD_3OD) δ 159.53 (dtd, J = 242.3, 71.3, 10.2 Hz), 143.00, 133.90 (tdd, J = 64.3, 10.2, 2.9 Hz), 122.11 (ddt, J = 64.8, 56.0, 7.7 Hz), 117.37 – 112.25 (m). ^{19}F NMR (376 MHz, CD_3OD) δ -116.39 – -123.23 (m). MS (ESI+): 189.00.

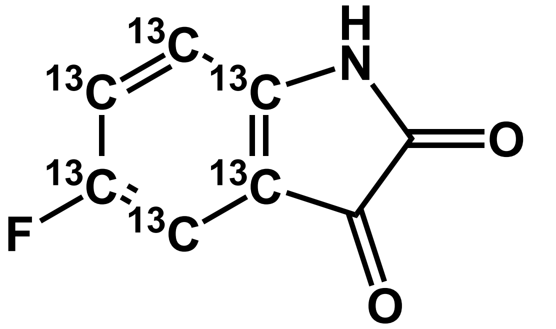

5-Fluoroisatin-(*phenyl*-$^{13}C_6$)

A two-necked flask equipped with a thermometer and a magnetic stir bar was charged with concentrated sulfuric acid (1.8 mL, 0.8 mL/mmol) and the solution was warmed to 80 °C in an oil

bath. The isonitrosoacetanilide product from the previous step (420 mg, 2.23 mmol, 1.0 equiv.) was added portionwise to the mixture while stirring and the deep red-violet solution was stirred at 80 °C for 30 minutes. The reaction mixture was cooled to room temperature over an ice bath and rapidly poured into a vigorously stirred mixture of 5:1 ice water/ethyl acetate. The organic phase was separated, and the aqueous phase was extracted twice with ethyl acetate. The combined red organic phases were dried over sodium sulfate, the solvent was removed under reduced pressure, and the crude product was dried *in vacuo* to afford the product as a red solid (286 mg, 1.67 mmol, 75% yield). ^1H NMR (400 MHz, CD_3CN) δ 9.02 (s, 1H), 7.66 – 7.47 (m, 1H), 7.27 – 7.05 (m, 1H), 6.78 (qd, J = 8.8, 4.3 Hz, 1H). ^{13}C NMR (101 MHz, CD_3CN) δ 159.87 (dtdd, J = 241.3, 73.0, 9.8, 2.0 Hz), 149.37 – 146.04 (m), 125.81 (dddt, J = 72.9, 59.1, 24.4, 5.3 Hz), 121.24 – 118.68 (m), 114.61 (dddd, J = 67.1, 59.1, 7.4, 4.0 Hz), 113.48 – 110.95 (m). ^{19}F NMR (376 MHz, CD_3CN) δ -121.14 – -122.42 (m).

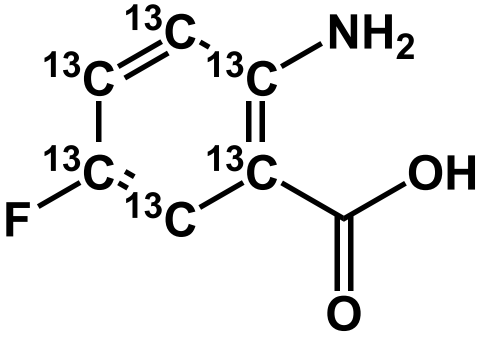

5-Fluoroanthranilic acid-(*phenyl*-$^{13}C_6$)

A three-necked round-bottom flask equipped with a magnetic stir bar, an addition funnel, and a thermometer was charged with 5-fluoroisatin-(*phenyl*-$^{13}C_6$) (286 mg, 1.67 mmol, 1.0 equiv.) and 3 M aqueous sodium hydroxide solution (1.11 mL, 0.67 mL/mmol, 2.0 equiv.). Hydrogen peroxide solution (30% in water; 0.334 mL, 0.2 mL/mmol, 2.0 equiv.) is added dropwise at 0 °C over 30 minutes. The solution was stirred for an additional 1 hour and the clear, pale yellow-orange reaction mixture was carefully acidified with vigorous stirring to a pH of 1 with 6 M hydrochloric, during which time the effervescing solution turns pale yellow and a beige solid precipitated. After an hour of stirring, the product is collected on a funnel and dried *in vacuo* over phosphorus pentoxide to afford the product as a beige solid (162 mg, 1.01 mmol, 60% yield). ^1H NMR (400 MHz, DMSO-d_6) δ 8.64 (s, 2H), 7.64 – 7.10 (m, 1H), 7.45 – 6.87 (m, 1H), 7.06 – 6.47 (m, 1H). ^{13}C NMR (101 MHz, $CDCl_3$) δ 156.32 – 150.89 (m), 149.75 – 146.22 (m), 125.64 – 121.40 (m),

118.04 (t, J = 57.7 Hz), 117.73 – 115.55 (m), 111.00 – 107.42 (m). ^{19}F NMR (376 MHz, $CDCl_3$) δ -128.20 (m). MS (ESI–): 159.83.

Section S3. Supplementary Figures and Tables

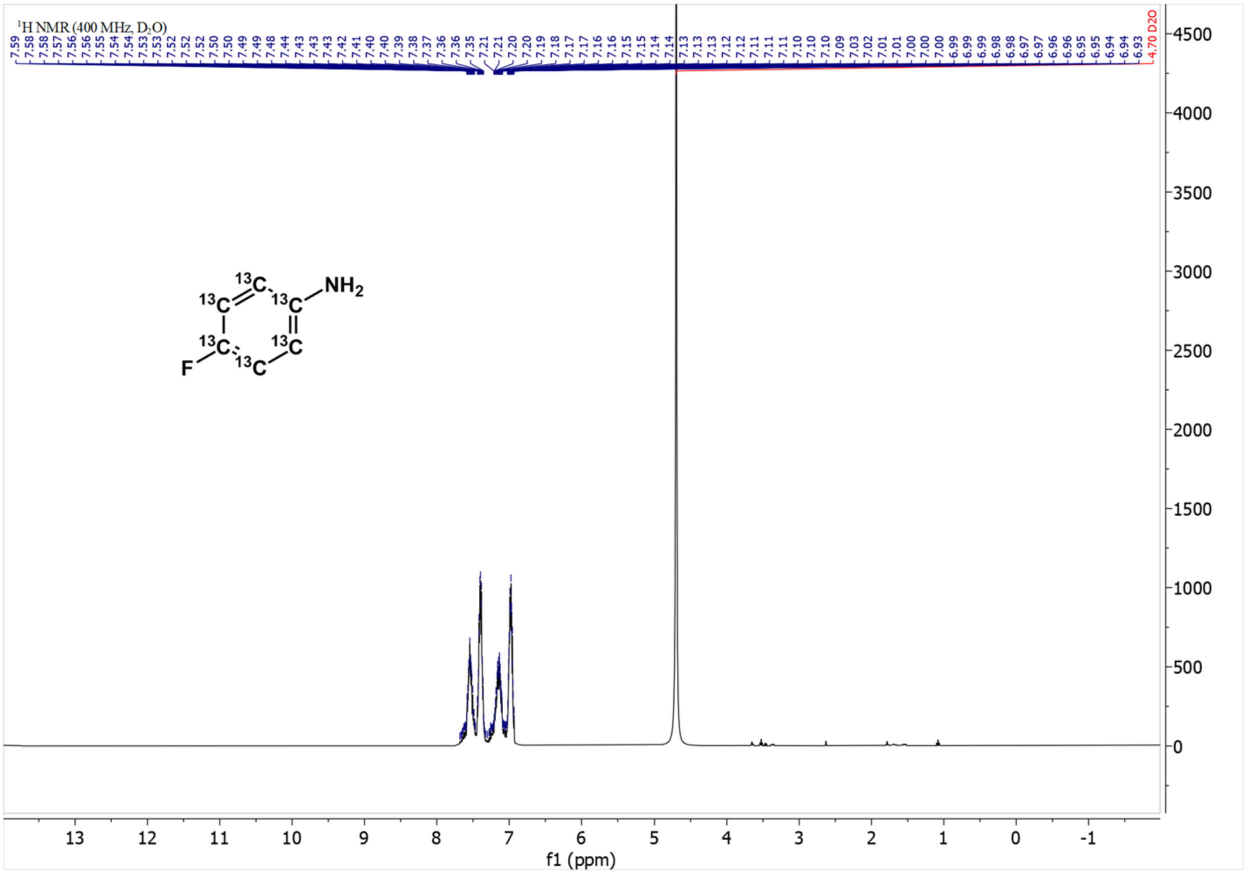

Figure S2. ^{1}H NMR spectrum of 4-fluoroaniline-$^{13}C_6$ hydrochloride.

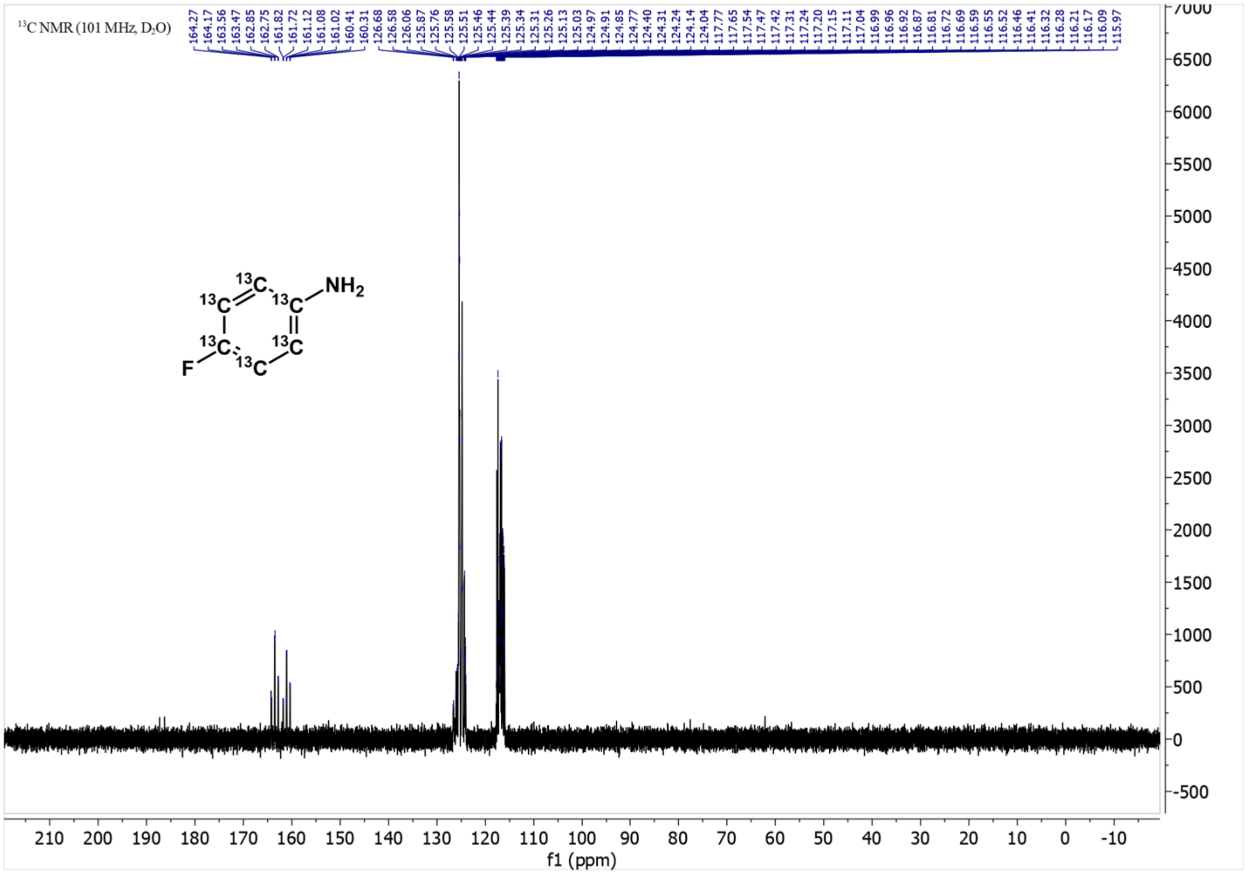

Figure S3. ^{13}C NMR spectrum of 4-fluoroaniline-$^{13}C_6$ hydrochloride.

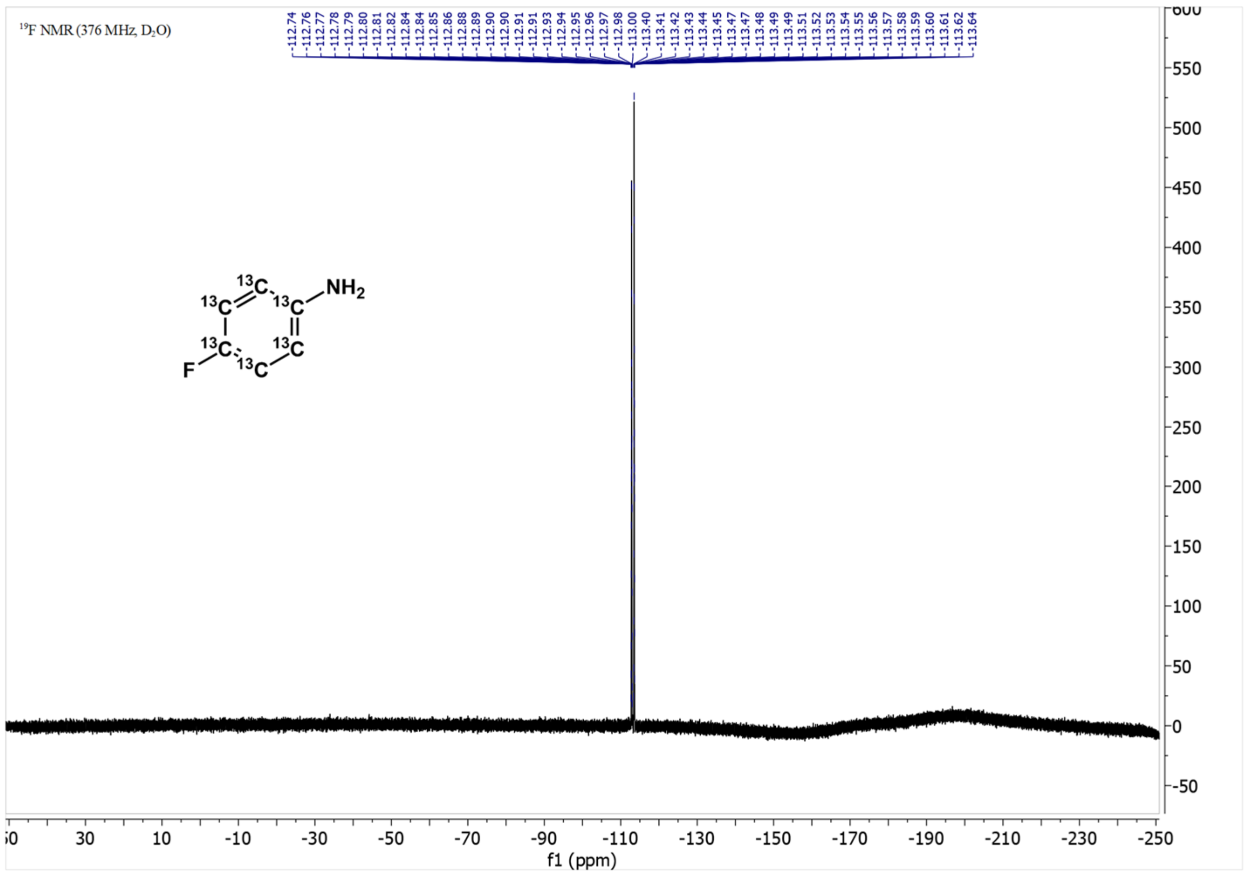

Figure S4. ^{19}F NMR spectrum of 4-fluoroaniline-$^{13}C_6$ hydrochloride.

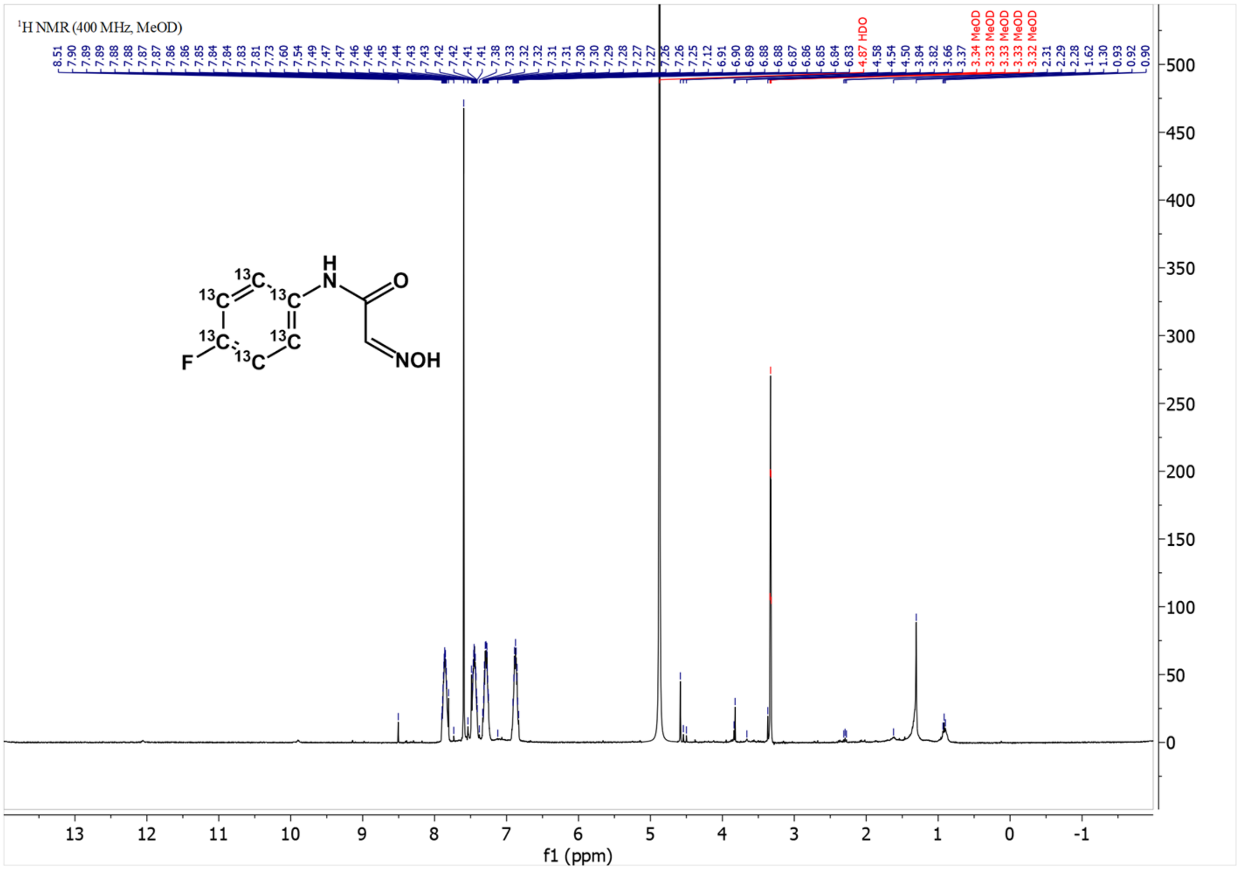

Figure S5. ^{1}H NMR spectrum of *N*-(4-fluorophenyl)-2-isonitrosoacetanilide-(*phenyl*-$^{13}C_6$).

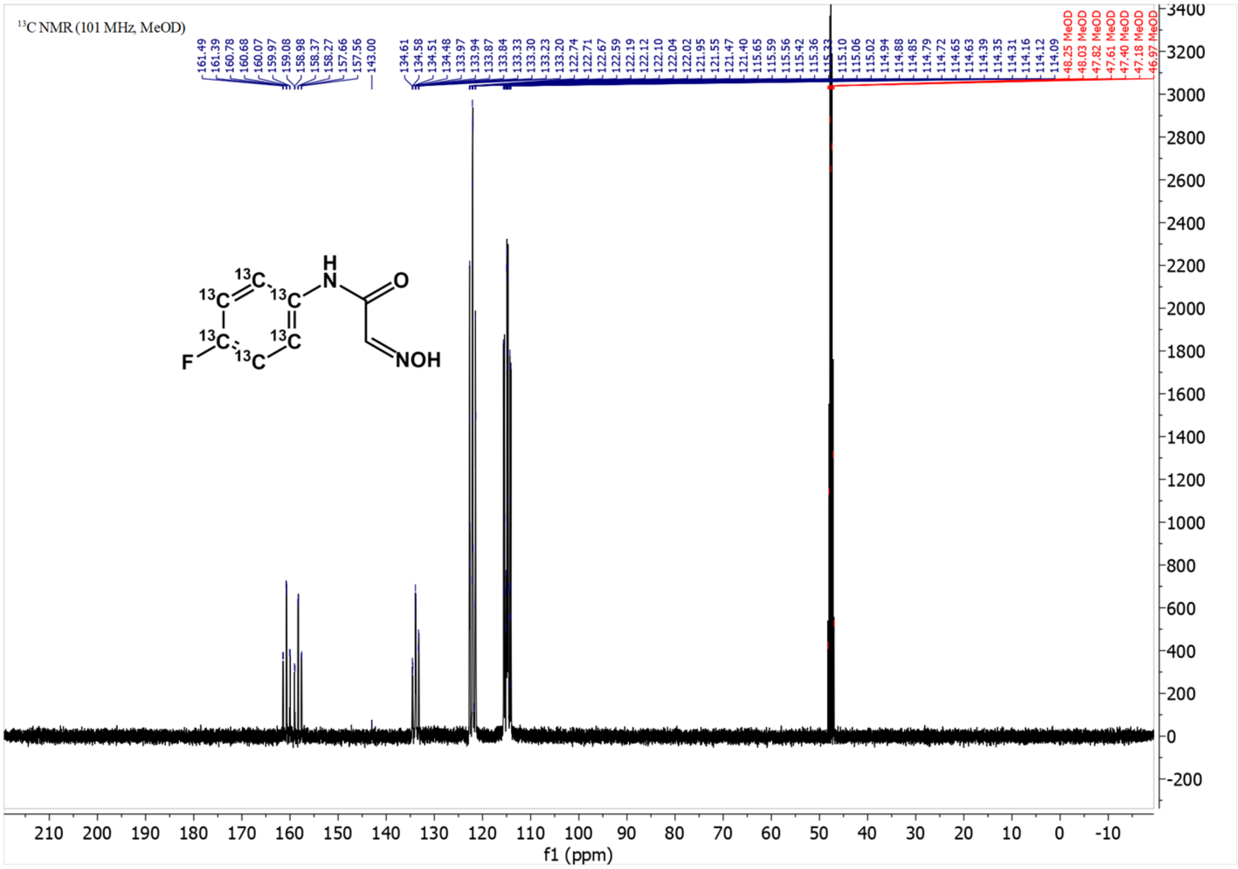

Figure S6. ^{13}C NMR spectrum of *N*-(4-fluorophenyl)-2-isonitrosoacetanilide-(*phenyl*-$^{13}C_6$).

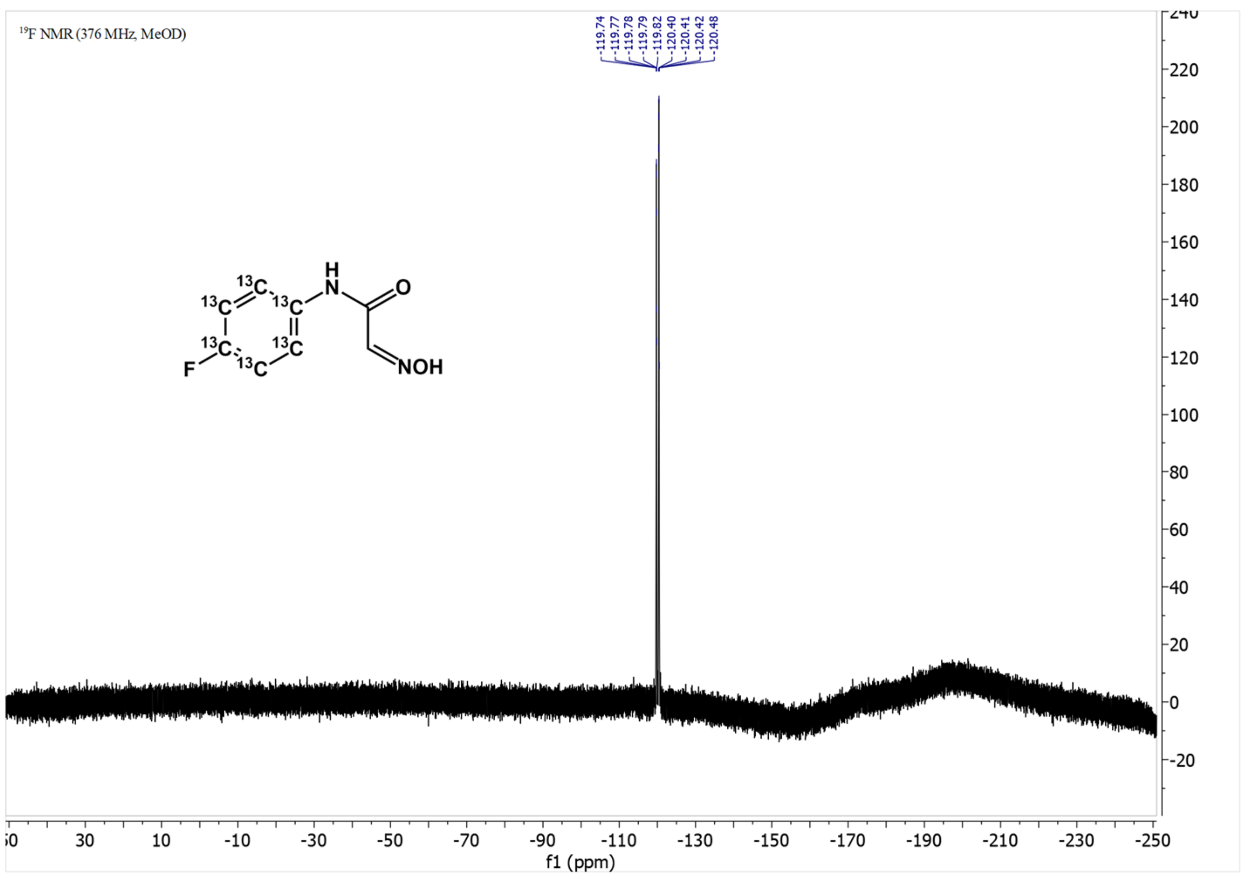

Figure S7. ^{19}F NMR spectrum of *N*-(4-fluorophenyl)-2-isonitrosoacetanilide-(*phenyl*-$^{13}C_6$).

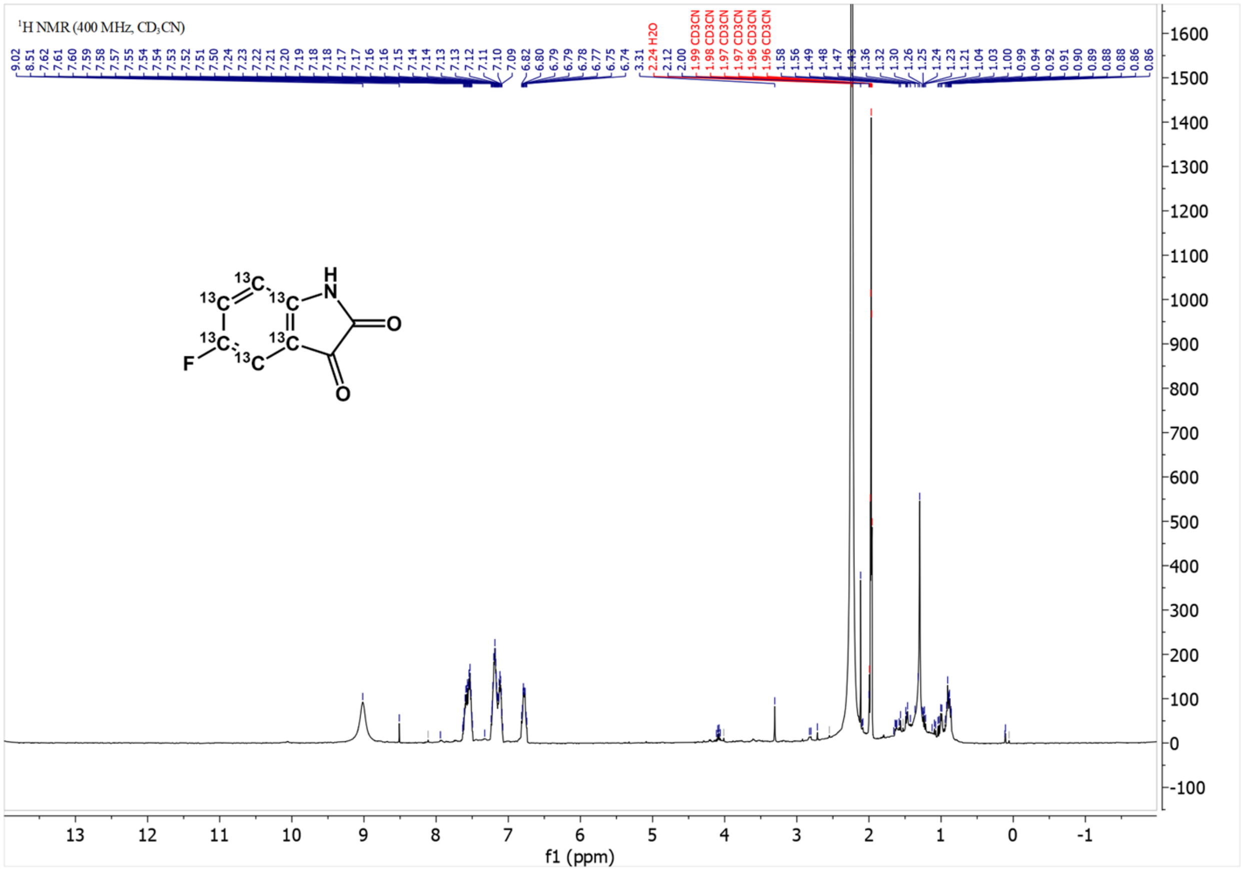

Figure S8. ^{1}H NMR spectrum of 5-fluoroisatin-(*phenyl*-$^{13}C_6$).

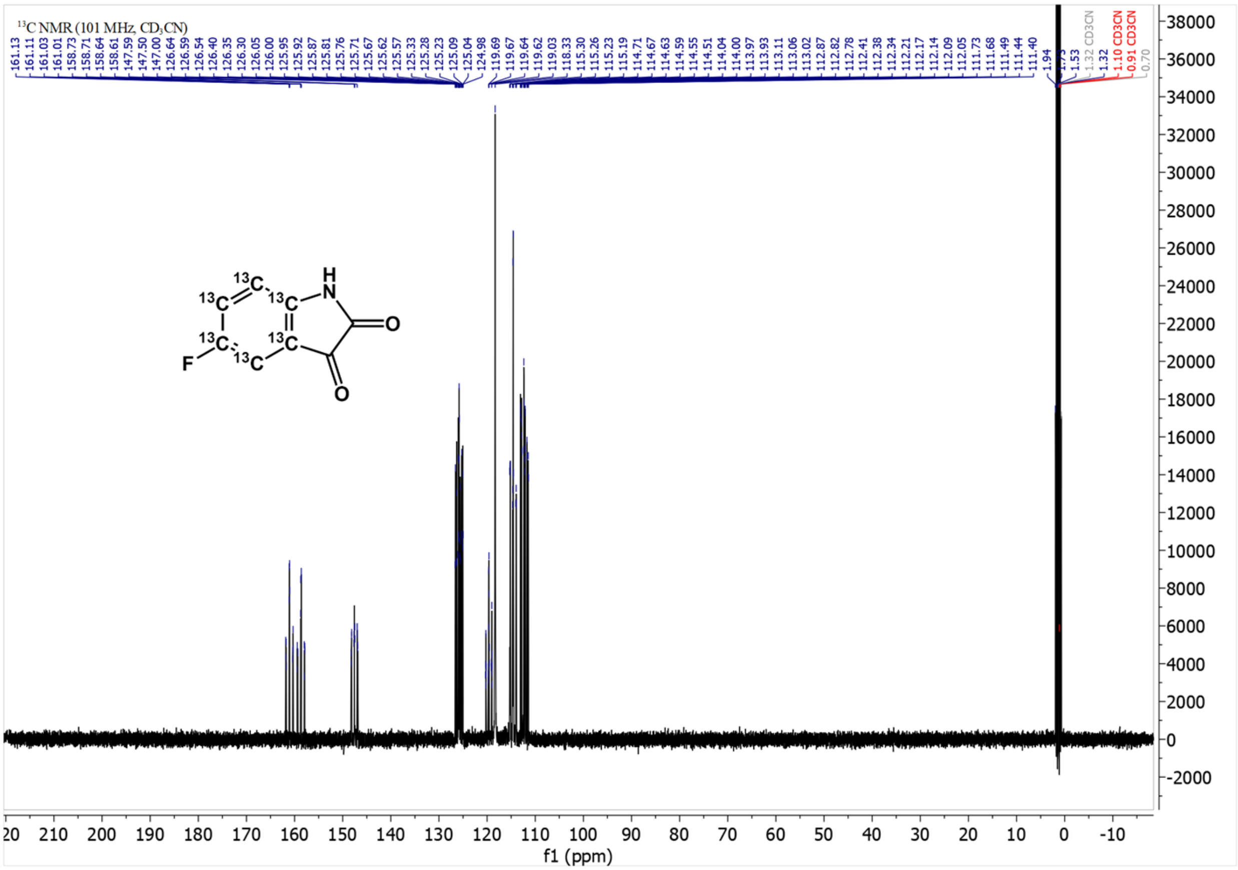

Figure S9. ^{13}C NMR spectrum of 5-fluoroisatin-(*phenyl*-$^{13}C_6$).

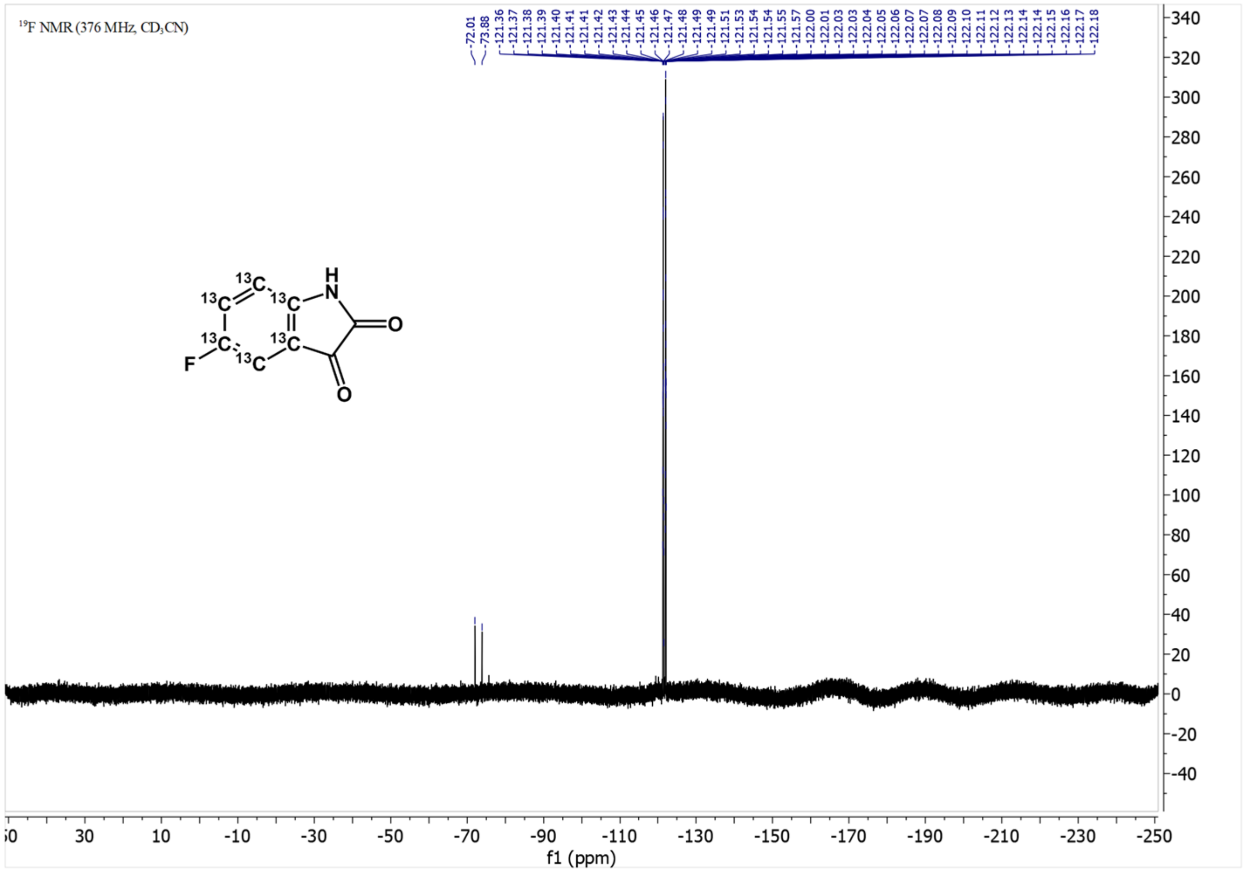

Figure S10. ^{19}F NMR spectrum of 5-fluoroisatin-(*phenyl*-$^{13}C_6$).

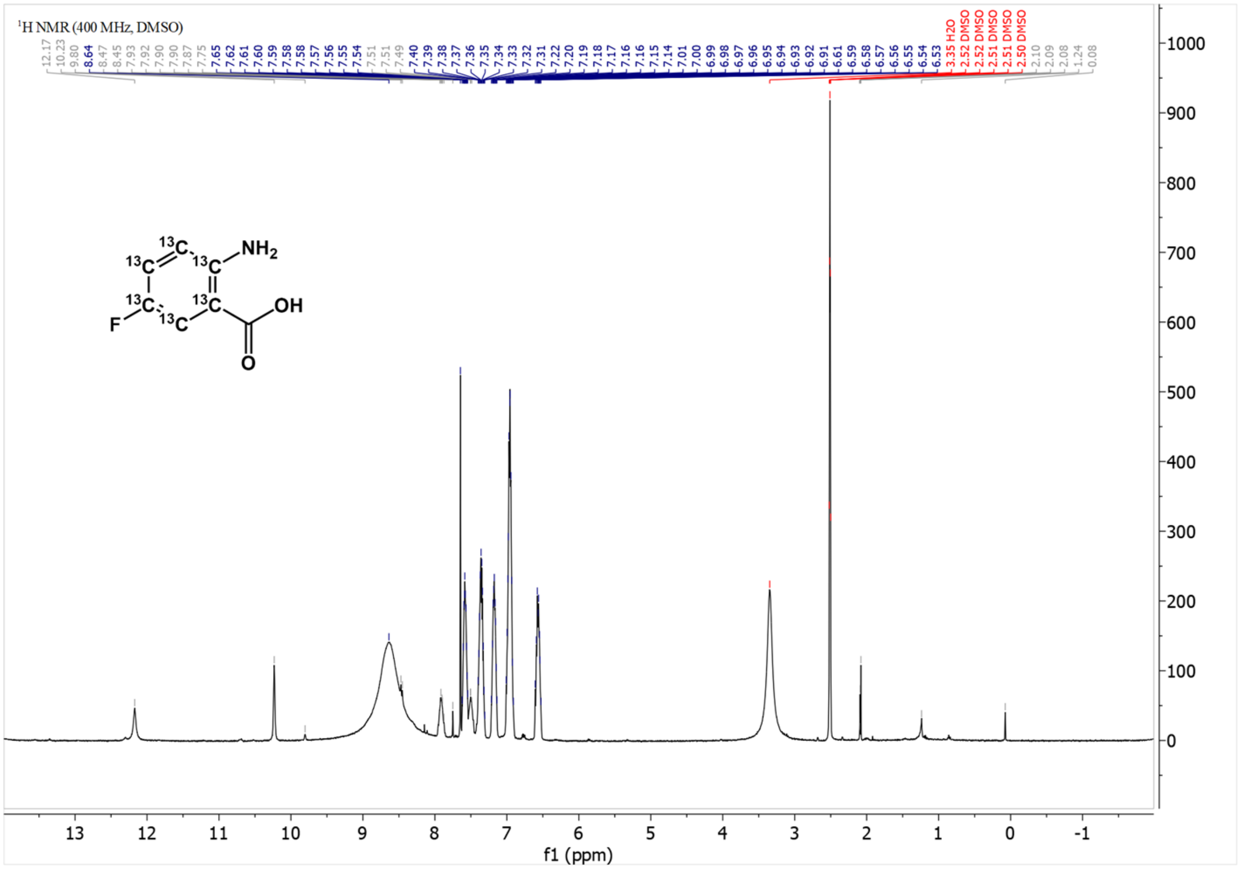

Figure S11. ^{1}H NMR spectrum of 5-fluoroanthranilic acid-(*phenyl*-$^{13}C_6$).

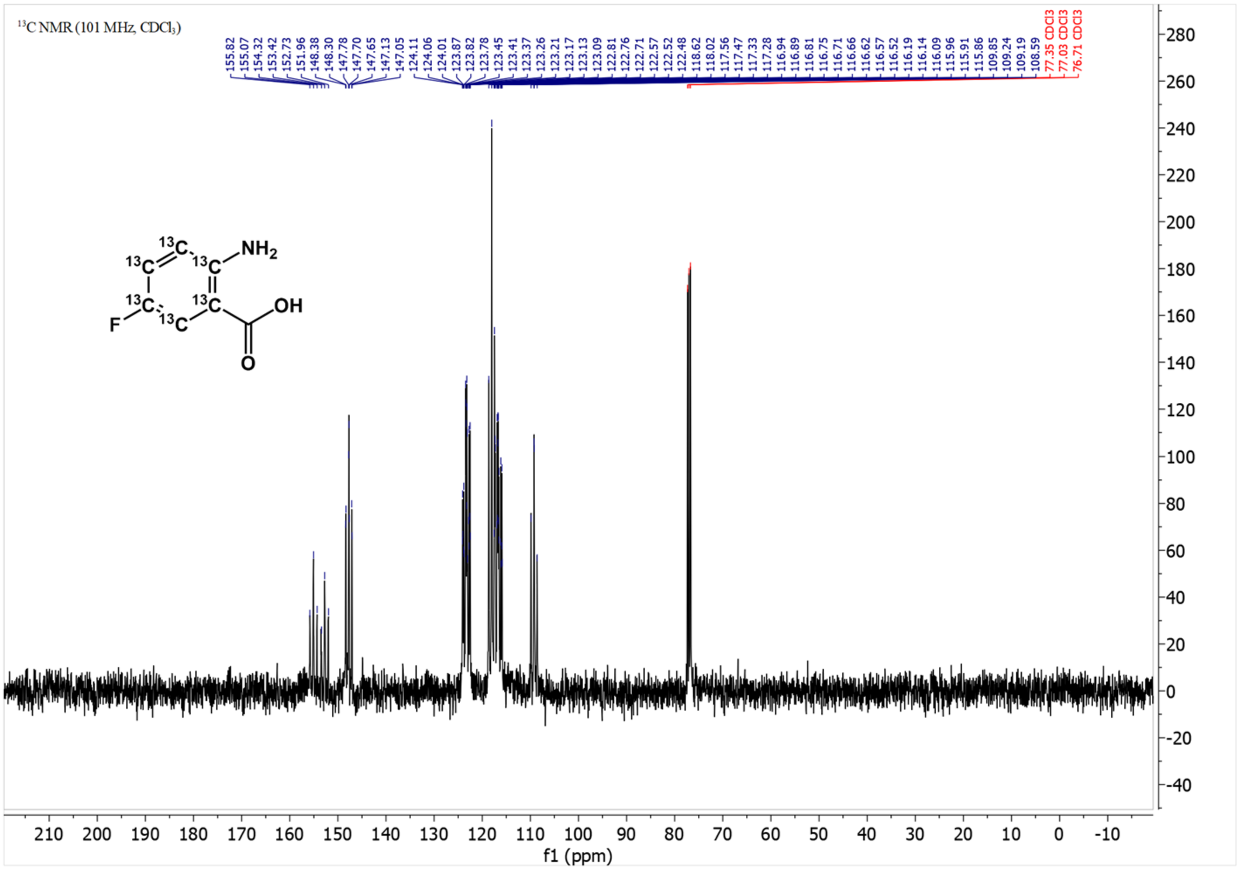

Figure S12. ^{13}C NMR spectrum of 5-fluoroanthranilic acid-(*phenyl*-$^{13}C_6$).

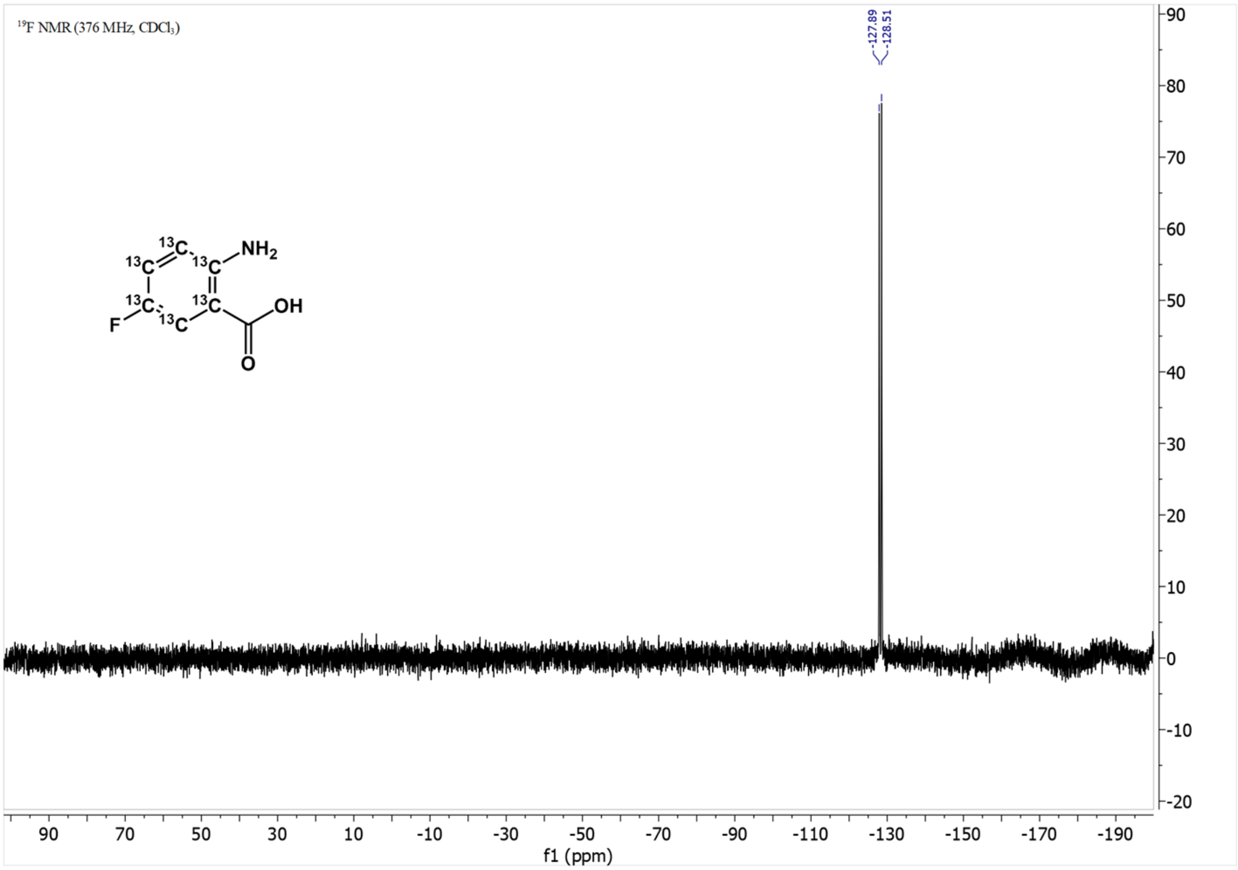

Figure S13. ^{19}F NMR spectrum of 5-fluoroanthranilic acid-(*phenyl*-$^{13}C_6$).

References

1 T. H. Kim, P. Mehrabi, Z. Ren, A. Sljoka, C. Ing, A. Bezginov, L. Ye, R. Pomès, R. S. Prosser and E. F. Pai, *Science*, 2017, **355**, eaag2355-39.

2 P. Mehrabi, C. D. Pietrantonio, T. H. Kim, A. Sljoka, K. Taverner, C. Ing, N. Kruglyak, R. Pomès, E. F. Pai and R. S. Prosser, *Journal of the American Chemical Society*, 2019, **141**, 11540–11556.

3 G. Bodenhausen and D. Ruben, *Chemical Physics Letters*, 1980, **69**, 185–189.

4 A. Boeszoermenyi, S. Chhabra, A. Dubey, D. L. Radeva, N. T. Burdzhiev, C. D. Chanev, O. I. Petrov, V. M. Gelev, M. Zhang, C. Anklin, H. Kovacs, G. Wagner, I. Kuprov, K. Takeuchi and H. Arthanari, *Nature methods*, 2019, **16**, 1–12.

5 N. J. Fowler, A. Sljoka and M. P. Williamson, *Nat Commun*, 2020, **11**, 6321.

6 A. Sljoka, in *ALLOSTERY: Methods and Protocols*, 2021, vol. 2253, pp. 61–75.

7 S. K. Huang, O. Almurad, R. J. Pejana, Z. A. Morrison, A. Pandey, L.-P. Picard, M. Nitz, A. Sljoka and R. S. Prosser, *Elife*, 2022, **11**, e73901.

8 S. K. Huang, A. Pandey, D. P. Tran, N. L. Villanueva, A. Kitao, R. K. Sunahara, A. Sljoka and R. S. Prosser, *Cell*, 2021, **184**, 1884-1894.e14.

9 S. K. Huang, L.-P. Picard, R. S. M. Rahmatullah, A. Pandey, N. V. Eps, R. K. Sunahara, O. P. Ernst, A. Sljoka and R. S. Prosser, *Nat Struct Mol Biol*, 2023, **30**, 502–511.

10 W. Whiteley, *Phys Biol*, 2005, **2**, S116-26.

11 A. Sljoka, in Sublinear Computation Paradigm, N. Katoh et al. (eds.), 2022, 337–367.

12 D. J. Jacobs, A. J. Rader, L. A. Kuhn and M. F. Thorpe, *Proteins Struct Funct Bioinform*, 2001, **44**, 150–165.
